# Supplementary material for: Towards Understanding Excited-State Properties of Organic Molecules Using Time-Resolved Soft X-ray Absorption Spectroscopy
Source: Int J Mol Sci. 2021 Dec 15;22(24):13463. doi: 10.3390/ijms222413463 (PMC8706469; doi:10.3390/ijms222413463)
Supplement: Supplementary file 1 [file ijms-22-13463-s001.zip › ijms-1473961-supplementary.pdf]

Supplemental information for

Towards understanding excited state properties of organic molecules using time-resolved soft X-ray absorption spectroscopy

Holger Stiel[1,3,*], Julia Braenzel[1,3], Adrian Jonas[1,2], Richard Gnewkow[1,2,4], Lisa Theresa Glöggler[1,2,#], Denny Sommer[3], Thomas Krist[6], Alexei Erko[5], Johannes Tümmler[1,3], Ioanna Mantouvalou[1,2,4]

[1)] *Berlin Laboratory for innovative X-ray technologies (BLiX), D-10623 Berlin, Germany*
[2)] *TU Berlin, Analytical X-Ray Physics, D-10623 Berlin, Germany*
[3)] *Max-Born-Institut für Nichtlineare Optik und Kurzzeitspektroskopie, D-12489 Berlin, Germany*
[4)] *Current affiliation: Helmholtz Zentrum Berlin, D-12489, Germany*
[5)] *IAP eV, D-12489 Berlin, Germany*
[6)] *Nano Optics Berlin GmbH, D-10627 Berlin, Germany*
[#] now at Physics Department, CERN, 1211 Geneva, Switzerland.
[*] *stiel@mbi-berlin.de*

Data acquisition and evaluation

For the sub-ns setup, the data evaluation is done in an automated Python routine. Each image is processed individually. First, an averaged background spectrum is subtracted. Usually, 200 dark images are collected every 2000 illuminated shots. The source image is then cropped and evaluated using a 2D Gaussian fit. The rows of the part containing the reference and sample spectra are summed up. The sample spectrum is then flipped and shifted to match the pixel axis, which becomes the energy axis after calibration. The NEXAFS spectrum is obtained by the natural logarithm of the ratio of the reference spectrum divided by the sample spectrum.

The energy axis is calibrated by assigning tabulated atomic emission lines of a known plasma spectrum, which optimally shows isolated emission lines throughout the measured energy range. Typically, a copper emission spectrum is utilized, resulting in an absolute error of the calibration between 0.2 eV and 0.5 eV. Alternatively, the spectra can be calibrated with known NEXAFS spectra from previous measurements or literature.

For the ps-setup, a single RZP optic, the Test RZP A9, is used (cp. Fig. 1) that focuses the soft X-ray spectrum onto the whole detector area. NEXAFS measurements with NiO sample at the ps-LPP were evaluated for 100 images and 100 background images. An exposure time of about 20 ms was applied that amounts to two laser pulses per measurement. The background images contained stray light originating from the beamline with the RZP being removed from the beam axis but at full laser–target interaction. The spectrum transmitted through the sample was recorded with a backside-illuminated CCD detector (see above) and corrected from background and straylight. It was found that the LPP source position fluctuation is decreasing the resolving power for longer integration times. This source pointing was determined to result from the ablation of the target material, which is as-of-yet unavoidable. As a consequence, shorter integration times are necessary, and for close to single laser pulse measurements, this still leads to a shift in the spectrum by a few pixels of the detector. Therefore, the binned spectra of single images needed a minor correction (few-pixel range) for the source position fluctuations

by applying a simple pixel shift routine on a high contrast with a spectral limited feature in the spectrum. Further, the single image series were spectrally calibrated using the analytical equation for the RZP and a parametric iteration of the RZP input angle to fit spectral references in the spectrum. Then, the image series were statistically evaluated (mean value of data series), and the NEXAFS was calculated.

Table S1 summarizes the experimental conditions for data acquisition using the sub-ns and ps-NEXAFS setups.

Table S1

Edge	#shots	#background images	Laser pulse energy (mJ)	Pulse duration (ps)
TAP C-K (ground state)	8000	800	120	500
TAP C-K (transient)	20000	2000	120	500
TAP N-K	5000	500	120	500
PIC C-K	200	10	120	1000
PIC N-K	200	10	150	1000
NiO Ni-L2	5000	500	140	1000
NiO Ni-L3	4000	400	140	1000
NiO O-K	4000	200	140	1000
NiO Ni-L2,L3	200	100	130	1.5

For reference purposes, the NEXAFS spectrum of the NiO sample has been measured at the SX700 beamline of PTB at BESSYII/Berlin. The step size amounted to 500 meV and the energy resolution was $E/\Delta E$ y 1000.

Sample preparation

The organic molecule samples were prepared using an effusion cell (OLED-40-2-SHM CreaTec Ltd.). The sample powder is placed inside a 200 mg quartz crucible, which is heated by a tungsten wire. A heating control unit (Eurotherm Type 3504) can be programmed to heat the sample in arbitrary heating scenarios, with 400 °C as the maximal temperature and 0.1 °C precision. The crucible has a plug to restrict the molecular beam, and a shutter is used for coating time control. Between the shutter and sample mount, a protection plate ensures that only one substrate is coated. The sample holder can accommodate five substrates and can be moved vertically to change the distance between crucible and substrate, allowing for a change in the growth rate. A quartz crystal sensor is integrated into the setup and connected with an STM-2 (INFICON) rate and thickness monitor. The whole setup operates at 10^{-6} mbar pressure.

TAP powder was purchased from Sigma-Aldrich and used without further purification. Using a small spatula, 35 mg of TAP is placed in the crucible of the effusion cell. The crucible is slowly heated to 210°C, which is 28°C above the melting point of TAP given by the Sigma-Aldrich data sheet. After reaching 210°C, one substrate is coated for about 20 min resulting in a TAP film thickness of about 200 nm. After one hour, the deposition rate drops so that three substrates can be coated consecutively. A larger amount of sample powder cannot be used to coat more substrates because part of the sample occasionally is blown out abruptly, leaving too little in the crucible to completely coat one substrate. Additionally, sometimes carbonization occurs, leaving a black film in the crucible. After the evaporation process, the cell is slowly cooled to room temperature, and the coated samples can be removed from the cell.

PIC powder was also purchased from Sigma-Aldrich and used without purification. Three samples were produced in three separated evaporation processes with 60 mg, 30 mg and 70 mg of material in the crucible. The evaporation temperature was set to 244°C, which is 29°C below the melting point given by the

manufacturer. Evaporation times of 15 min, 17 min and 5 min resulted in film thicknesses of 270 nm, 190 nm and 80 nm, respectively. In the case of the PIC molecule, no carbonization occurred.

The **NiO** samples were prepared on 200 nm thick Si_3N_4 windows (3x3mm) by depositing Ni using a reactive electron beam evaporation in an oxygen environment. The oxygen partial pressure was about $3 \cdot 10^{-4}$ mbar at a Ni deposition rate of 0.5 $Å \cdot s^{-1}$. The layer thickness was monitored by a quartz oscillator during the process. The stoichiometry of the NiO layer was determined after preparation by energy-dispersive X-ray spectroscopy (EDX) using the JSM-6400F von JEOL with an acceleration voltage of 4 keV (see Fig.S1)

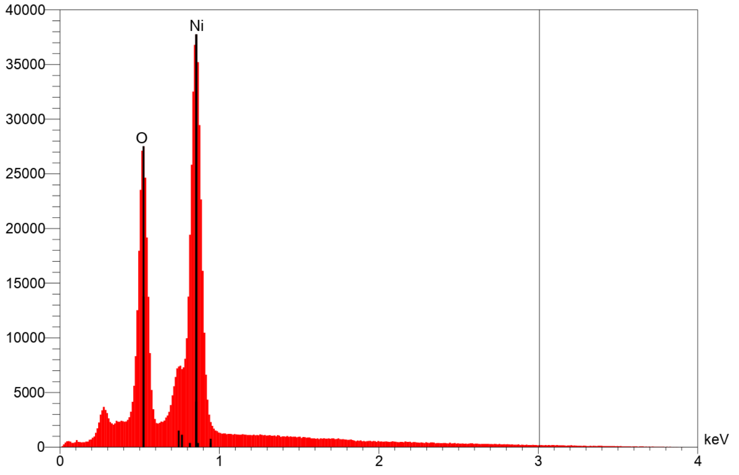

Quantitative Ergebnisse

Elt	Line	Int	Fehler	K	Kr	Gew.-%	Atom-%	Summenformel	Oxid-%
O	Ka	289.8	53.8121	0.2691	0.2574	22.55	51.66	1.1413	0.00
Ni	La	429.6	53.8121	0.7309	0.6990	77.45	48.34	0.9026	0.00
				1.0000	0.9565	100.00	100.00		0.00

Figure S1: Results from the EDX measurement of the NiO sample.

Thickness and homogeneity of the sample were checked after preparation by an AFM (see Fig.S2)

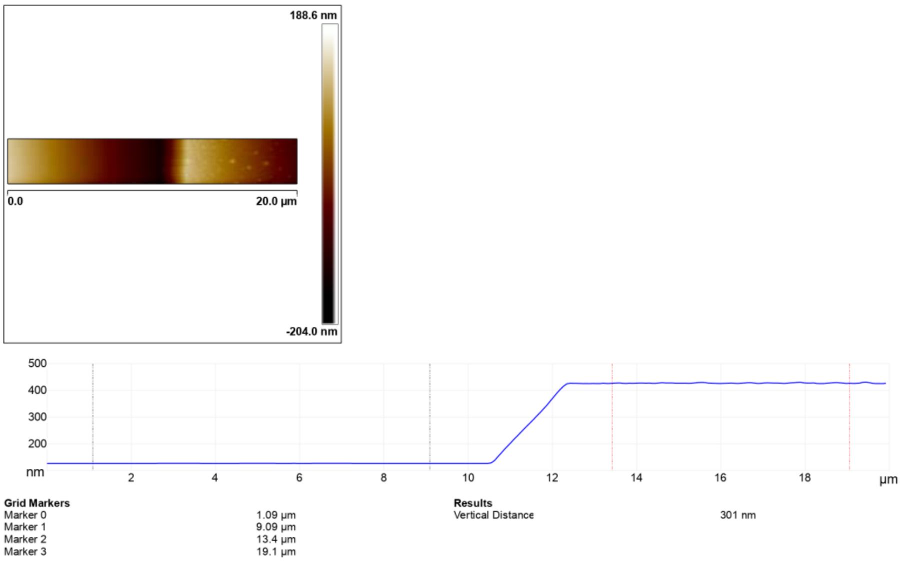

Figure S2: Results from the AFM measurement of the NiO sample.

Sample characterization

UV/Vis spectroscopy

The UV/VIS spectra were measured using the Lambda 900 spectrometer by PerkinElmer. The absorption spectra (Fig. 6 and 7) were collected with a data interval of 0.5 nm and a scan rate of 141.2 nm/min.

Sample thickness and homogeneity

During the evaporation process, the sample thickness has been monitored by a quartz oscillator.

The thickness of the thin organic films have be measured spatially resolved over the full size of the sample using a table top EUV spectrometer operating in transmission mode at 18.9 nm [1]. The EUV spectrometer is based on a laser-produced plasma source (1064 nm, 3 ns, 200 mJ, Mo-target). Film thickness was determined by a procedure described in [1]. The data shown in Fig.6 (inset) were collected using 10 laser shots for both sample transmission and reference and a background correction.

Atomic force microscopy (AFM)

We used a Dimension Icon AFM by Bruker. The AFM was operated in tapping mode. Mapping the entire sample area with AFM requires a lot of effort, as the method is considerably time-consuming. For this reason, only sample subsets in the range of few μm^2 have been imaged.

Reference

1. Braenzel, J.; Pratsch, C.; Hilz, P.; Kreuzer, C.; Schnürer, M.; Stiel, H.; Sandner, W., Note: Thickness determination of freestanding ultra-thin foils using a table top laboratory extreme ultraviolet source. *Review of Scientific Instruments* **2013,** 84, (5), 3.
